# Evaluation of Absolute Lymphocyte Count at Diagnosis and Mortality Among Patients With Localized Bone or Soft Tissue Sarcoma

**DOI:** 10.1001/jamanetworkopen.2021.0845

**Published:** 2021-03-05

**Authors:** Ryan Brewster, Natasha Purington, Solomon Henry, Douglas Wood, Kristen Ganjoo, Nam Bui

**Affiliations:** 1 Stanford University School of Medicine, Stanford, California; 2 Quantitative Sciences Unit, Stanford University School of Medicine, Stanford, California; 3 Deprtment of Biomedical Data Science, Stanford University, Stanford, California; 4 Department of Medicine (Oncology), Stanford University School of Medicine, Stanford, California

## Abstract

**Importance:**

Host-related immune factors have been implicated in the development and progression of diverse malignant neoplasms. Identifying associations between immunologic laboratory parameters and overall survival may inform novel prognostic biomarkers and mechanisms of antitumor immunity in localized bone and soft tissue sarcoma.

**Objective:**

To assess whether lymphopenia at diagnosis is associated with overall survival among patients with localized bone and soft tissue sarcoma.

**Design, Setting, and Participants:**

This retrospective cohort study analyzed patients from the Stanford Cancer Institute with localized bone and soft tissue sarcoma between September 1, 1998, and November 1, 2018. Patients were included if laboratory values were available within 60 days of diagnosis and, if applicable, prior to the initiation of chemotherapy and/or radiotherapy. Statistical analysis was performed from January 1, 2019, to November 1, 2020.

**Exposures:**

Absolute lymphocyte count within 60 days of diagnosis and antimicrobial exposure, defined by the number of antimicrobial agent prescriptions and the cumulative duration of antimicrobial administration within 60 days of diagnosis.

**Main Outcomes and Measures:**

The association between minimum absolute lymphocyte count at diagnosis and 5-year overall survival probability was characterized with the Kaplan-Meier method and multivariate Cox proportional hazards regression models. Multivariable logistic regressions were fitted to evaluate whether patients with lymphopenia were at greater risk of increased antimicrobial exposure.

**Results:**

Among 634 patients, the median age at diagnosis was 53.7 years (interquartile range, 37.5-66.8 years), and 290 patients (45.7%) were women, with a 5-year survival probability of 67.9%. There was a significant inverse association between lymphopenia at diagnosis and overall survival (hazard ratio [HR], 1.82; 95% CI, 1.39-1.40), resulting in a 13.5% 5-year survival probability difference compared with patients who did not have lymphopenia at diagnosis (60.2% vs 73.7% for those who never had lymphopenia). In addition, poorer survival was observed with higher-grade lymphopenia (grades 3 and 4: HR, 2.44; 95% CI, 1.68-3.55; grades 1 and 2: HR, 1.60; 95% CI, 1.18-2.18). In an exploratory analysis, patients with increased antibiotic exposure were more likely to have lymphopenia (odds ratio, 1.96; 95% CI, 1.26-3.07 for total number of antimicrobial agents; odds ratio, 1.70; 95% CI, 1.10-2.57 for antimicrobial duration) than antimicrobial-naive patients.

**Conclusions and Relevance:**

This study suggests that an abnormally low absolute lymphocyte count at diagnosis is associated with higher mortality among patients with localized bone and soft tissue sarcoma; therefore, lymphopenia may serve as a reliable prognostic biomarker. Potential mechanisms associated with host immunity and overall survival include a suppressed antitumor response and increased infectious complications, which merit future investigation.

## Key Points

**Question:**

Is there an association between lymphopenia at diagnosis and overall mortality among patients with localized bone and soft tissue sarcoma?

**Findings:**

In this cohort study of 634 patients with localized bone and soft tissue sarcoma, peripheral lymphocyte counts at diagnosis were significantly inversely associated with overall mortality.

**Meaning:**

This study suggests that absolute lymphocyte count may constitute a reliable prognostic biomarker for patients with bone and soft tissue sarcoma; however, further research is needed to define the mechanistic role of host antitumor immunity and infectious complications.

## Teaser

This cohort study assesses whether lymphopenia at diagnosis is associated with overall survival among patients with localized bone and soft tissue sarcoma.

## Introduction

Sarcomas constitute a rare but heterogenous group of malignant neoplasms originating from mesenchymal tissue. Despite advances in diagnostic and therapeutic modalities, outcomes among patients with high-grade sarcoma have remained poor.^1^ It is critical to validate novel biomarkers to stratify high-risk patients who may benefit from earlier aggressive therapeutic regimens.

The immunosurveillance theory posits that lymphocytes play a protective role in detecting and targeting malignant cells.^2,3^ This theory has generated substantial interest in developing immunotherapies for sarcomas, with promising results among a subset of patients.^4^ Furthermore, reports indicate that lymphopenia before treatment initiation is associated with higher mortality across diverse cancer types.^5^

In this retrospective cohort study of 634 patients treated at the Stanford Cancer Institute in Stanford, California, between 1998 and 2017, we evaluated the prognostic value of lymphopenia at diagnosis for patients with localized bone and soft tissue sarcoma and assessed the association of infectious complications as measured by antimicrobial exposure with lymphopenia.

## Methods

### Data and Study Cohort

We identified 634 eligible patients with a diagnosis of bone or soft tissue sarcoma who received a diagnosis at the Stanford Cancer Institute between September 1, 1998, and November 1, 2018, using a retrospective analysis within the Stanford Cancer Institute Research Database. Patients were included if they received a formal diagnosis of sarcoma at Stanford Cancer Institute and had laboratory values measured within 60 days of date of diagnosis. Patients with metastatic disease at diagnosis were excluded. This study was approved by the Stanford University institutional review board, which granted a waiver of consent because the research involved no more than minimal risk to participants and procedures were in place to protect confidentiality. This study followed the Strengthening the Reporting of Observational Studies in Epidemiology (STROBE) reporting guideline.

### Outcome and Exposure Definitions

Patients were classified as ever having lymphopenia if they had an absolute lymphocyte count (ALC) value less than 1000/µL (to convert to ×10^9^ cell per liter, multiply by 0.001) within 60 days of the date of sarcoma diagnosis and before the start of chemotherapy and/or radiotherapy. The minimum ALC refers to the lowest ALC value among all those measured during the aforementioned timeline. For patients designated as ever having lymphopenia, minimum ALC was graded in accordance with the Common Terminology Criteria for Adverse Events.^6^

### Exploratory Analysis of Antibiotic Exposure and Lymphopenia

We evaluated the association between use of antimicrobial agents and lymphopenia. Antibiotics and antifungals of interest were considered if orders were recorded within 60 days of diagnosis and prior to the initiation of chemotherapy and/or radiotherapy, if given. Exposure was quantified as both (1) the total number of unique antibiotics and antifungal prescriptions and (2) the total duration in nonoverlapping days receiving antibiotics and antifungals. Any patient who did not survive beyond 150 days after diagnosis was excluded from analysis. For patients receiving antimicrobials, median values were used as thresholds to delineate the subgroups (0, 1-3, and ≥4 antimicrobial agents and 0, 1-3, and ≥4 total days of antimicrobial administration).

### Statistical Analysis

Statistical analysis was performed from January 1, 2019, to November 1, 2020. The Kaplan-Meier method was used to estimate survival curves stratified on the basis of lymphopenic status or antibiotic exposure. Multivariate Cox proportional hazards regression models were generated to evaluate the association between minimum ALC and overall mortality. We adjusted for potential explanatory variables, including minimum white blood cell count, minimum absolute neutrophil count, tumor grade, and age at diagnosis. The asociations between lymphopenia and antibiotic exposure was assessed with multivariate logistic regression models, adjusted for tumor grade and age at diagnosis. All statistical analyses were performed with the use of R, version 1.1.463. *P* = .05 was defined as the threshold for statistical significance.

## Results

### Patient Characteristics

Between 1998 and 2017, a total of 2240 patients received a diagnosis of sarcoma at the Stanford Cancer Center, and 634 met the inclusion criteria. The median age at diagnosis was 53.7 years (interquartile range [IQR], 37.5-66.8 years), and 290 patients (45.7%) were women (Table 1). At diagnosis, the median value for ALC was 1120/µL (IQR, 700-1610/µL), the median white blood cell count was 6600/µL (IQR, 5200-7980/µL [to convert to ×10^9^ cells per liter, multiply by 0.001]), and the median absolute neutrophil count was 4610/µL (IQR, 3490-6190/µL [to convert to ×10^9^ cells per liter, multiply by 0.001]). A total of 281 patients (44.3%) ever had lymphopenia within 60 days of diagnosis. Five-year survival probability was 67.9%. The most common histologic subtypes were pleomorphic sarcoma (121 [19.1%]), liposarcoma (94 [14.8%]), and chondrosarcoma (65 [10.3%]).

**Table 1.** Characteristics of Patients

Characteristic	Patients, No. (%)		
	All (N = 634)	Lymphopenia	
		Ever (n = 281)	Never (n = 353)
Age at diagnosis, median (IQR), y	53.7 (37.5-66.8)	55.1 (31.0-68.3)	53.2 (38.3-65.3)
Women	290 (45.7)	122 (43.4)	168 (47.6)
Histologic subtype			
Chondrosarcoma	65 (10.3)	33 (11.7)	32 (9.1)
Ewing sarcoma	31 (4.9)	19 (6.8)	12 (3.4)
Fibromyxosarcoma	28 (4.4)	4 (1.4)	24 (6.8)
Giant cell sarcoma	32 (5.0)	13 (4.6)	19 (5.4)
Leiomyosarcoma	62 (9.8)	35 (12.5)	27 (7.6)
Liposarcoma	94 (14.8)	35 (12.5)	59 (16.7)
Pleomorphic sarcoma	121 (19.1)	53 (18.9)	68 (19.3)
Synovial sarcoma	36 (5.7)	13 (4.6)	23 (6.5)
Osteosarcoma	33 (5.2)	16 (5.7)	17 (4.8)
Rhabdomyosarcoma	30 (4.7)	16 (5.7)	14 (4.0)
Other	111 (17.5)	44 (15.7)	58 (16.4)
5-y survival probability, %	67.9	60.2	73.7
Grade			
1	88 (13.9)	38 (13.5)	50 (14.2)
2	81 (12.8)	32 (11.4)	49 (13.9)
3	85 (13.4)	38 (13.5)	47 (13.3)
4	163 (25.7)	72 (25.6)	91 (25.8)
Unknown	217 (34.2)	101 (35.9)	116 (32.9)
Laboratory test values at diagnosis, median (IQR), /µL			
ALC	1.12 (0.70-1.61)	0.66 (0.41-0.83)	1.56 (1.34-1.99)
WBC	6.60 (5.20-7.98)	5.60 (4.30-7.10)	7.20 (5.90-8.50)
ANC	4.61 (3.49-6.19)	4.42 (3.00-5.98)	4.71 (3.82-6.28)

Abbreviations: ALC, absolute lymphocyte count; ANC, absolute neutrophil count; IQR, interquartile range; WBC, white blood cell count.

SI conversion factors: To convert ALC, ANC, and WBC to ×10^9^/L, multiply by 0.001.

### Association of ALC With Overall Survival

The minimum ALC ranged from 60 to 5490/µL (median, 1120/µL). On multivariate analysis, patients who had ever had lymphopenia had a worse survival after adjusting for minimum white blood cell count, minimum absolute neutrophil count, tumor grade, and age at diagnosis (hazard ratio [HR], 1.82; 95% CI, 1.39-2.40) (Figure, A, and eTable 1 in the Supplement). This corresponded to a 60.2% 5-year survival probability compared with 73.7% for patients who never had lymphopenia. Grade 3 or 4 lymphopenia were associated with poorer outcomes (HR, 2.44; 95% CI, 1.68-3.55) than grade 1 or 2 lymphopenia (HR, 1.60; 95% CI, 1.18-2.18) (Figure, B, and eTable 2 in the Supplement). There was no significant association between ever having leukopenia (HR, 0.61; 95% CI, 0.33-1.12) or ever having neutropenia (HR, 0.58; 95% CI, 0.20-1.68) and survival.

**Figure.**
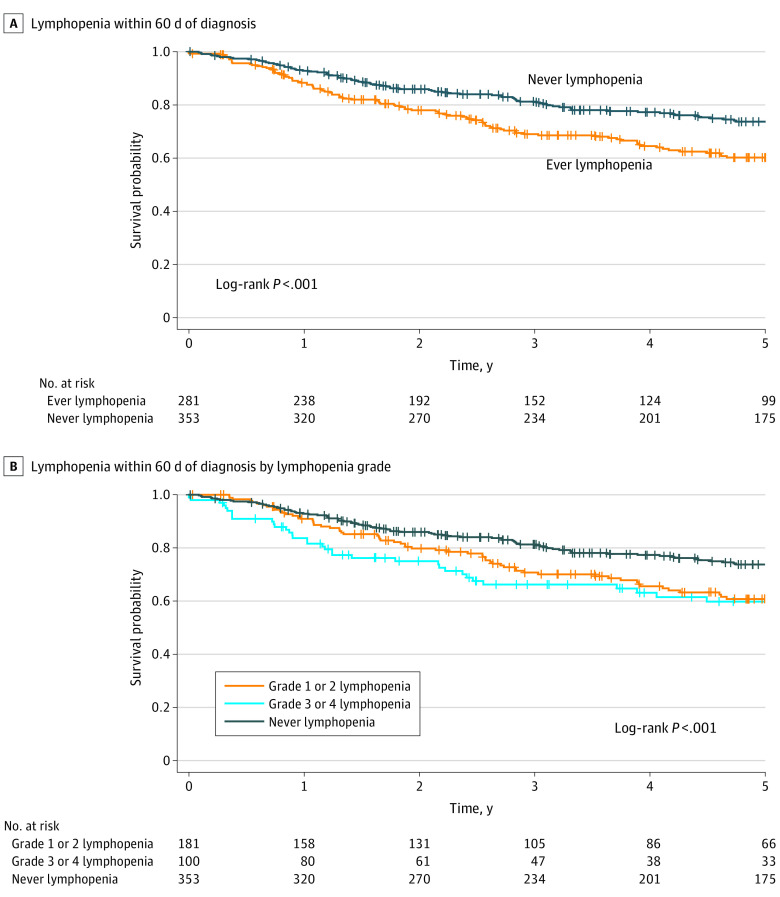
Association of Overall Survival With Lymphopenia Within 60 Days of Diagnosis Overall and by Lymphopenia Grade

### Antibiotic Exposure and Lymphopenia

A total of 302 of 602 patients (50.2%) did not receive any antimicrobials (Table 2). A total of 164 of 602 patients (27.2%) received 1 to 3 antimicrobial agents, and 136 of 602 patients (22.6%) received 4 or more distinct antimicrobial agents. A total of 163 of 602 patients (27.1%) had 1 to 3 days of antimicrobial exposure, and 133 of 602 patients (22.1%) had 4 or more days of antimicrobial exposure. There was no association between any amount of antimicrobial exposure and lymphopenia (odds ratio, 1.15; 95% CI, 0.82-1.59). However, on subgroup analysis, patients with 4 or more antimicrobial regimens were significantly more likely than antimicrobial-naive patients to have lymphopenia (odds ratio, 1.96; 95% CI, 1.26-3.07) (Table 2). Furthermore, patients whose cumulative duration of antimicrobial treatment was 4 or more days were also significantly more likely to have lymphopenia (OR, 1.70; 95% CI, 1.10-2.57)

**Table 2.** Multivariate Logistic Regression Models of the Association Between Antimicrobial Exposures and Lymphopenia

Antimicrobial exposure	Patients, No.	Odds ratio (95% CI)	*P* value
Antimicrobial agents, No.^a^			
0	302	1 [Reference]	
1-3	164	0.78 (0.53-1.12)	.18
≥4	136	1.96 (1.26-3.07)	.006
Duration of antimicrobial exposure, d^a^			
0	306	1 [Reference]	
1-3	163	0.80 (0.54-1.17)	.25
≥4	133	1.70 (1.10-2.57)	.005

^a^ Adjusted for tumor grade and age at diagnosis.

## Discussion

To our knowledge, this is the first report to identify lymphopenia at diagnosis of localized sarcoma as an independent prognostic biomarker. Among sarcomas, lymphopenia prior to initiating systemic therapy has been shown to correlate with poor survival among patients with advanced disease.^5^ Similarly, increased rates of recurrence and mortality after surgery are observed among patients with preoperative lymphopenia.^7^ Antitumor immunity relies on the recruitment of specific immune compartments to the tumor microenvironment. It is subject to complex autoregulation by proinflammatory molecules (eg, tumor-infiltrating lymphocytes) and anti-inflammatory molecules (eg, regulatory T cells and tumor-associated macrophages). The detection of tumor-infiltrating lymphocytes in retrospective histopathologic analysis corresponds to improved prognosis in various solid tumors and sarcoma subtypes.^8,9,10^ Pending further mechanistic insights, it is possible that a low peripheral lymphocyte count at sarcoma diagnosis limits the ability to mount a durable antitumor response, which may be associated with the poorer outcomes observed among patients with lymphopenia.

Our exploratory analysis of antimicrobial exposure suggests another possible consequence of lymphopenia. Opportunistic infections are a principal cause of mortality among patients with cancer.^11^ They have been associated with lymphocyte depletion, typically in the context of treatment-associated toxic effects.^12,13^ For example, lymphopenia secondary to chemotherapy in breast cancer has been shown to significantly increase the risk for *Pneumocystis* pneumonia.^14,15,16^ Thus, the poor outcomes observed among patients with sarcoma with lymphopenia at diagnosis may be partially explained by higher rates of infectious complications even prior to the initiation of therapy.

### Limitations

There are limitations to our study. First, this was a single-center observational cohort study; larger-scale studies are needed to assess the generalizability of our findings. In addition, we were unable to control for all possible confounding variables, and complete laboratory and clinical features were not available across the study population. There were also limitations with the method for evaluating antimicrobial exposure. Without hospital admission data, we approximated the burden of infections on the basis of antibiotic prescribing patterns, which do not exclude prophylactic indications or accurately capture the severity of infections. Ultimately, any suggestion that antibiotic exposure is associated with lower survival would need to be explored in future analyses.

## Conclusions

The findings of this study suggest that the ALC at diagnosis is inversely associated with mortality among patients with localized sarcoma and may potentially be used as a prognostic biomarker. Future studies should further evaluate clinical mediators of lymphopenia as well as the correlation between peripheral ALC and recruitment of tumor-infiltrating lymphocytes and other immune compartments to the tumor microenvironment. Altogether, these results add to the emerging understanding of host immunity in sarcoma and other solid tumor malignant neoplasms and suggest the powerful role of the native immune system in regulating cancer progression.

## Notes

Supplement. **eTable 1.** Multivariate Cox Regression Analysis of the Association Between Minimum ALC and Overall Survival **eTable 2.** Multivariate Cox Regression Analysis of the Association Between Minimum ALC, Stratified by Lymphopenia Grade, and Overall Survival

## References

[1] Amankwah EK, Conley AP, Reed DR. Epidemiology and therapies for metastatic sarcoma. Clin Epidemiol. 2013;5:147-162. doi:10.2147/CLEP.S28390 23700373 PMC3660127

[2] Sakaguchi S, Yamaguchi T, Nomura T, Ono M. Regulatory T cells and immune tolerance. Cell. 2008;133(5):775-787. doi:10.1016/j.cell.2008.05.009 18510923

[3] Schreiber RD, Old LJ, Smyth MJ. Cancer immunoediting: integrating immunity’s roles in cancer suppression and promotion. Science. 2011;331(6024):1565-1570. doi:10.1126/science.1203486 21436444

[4] Ayodele O, Razak ARA. Immunotherapy in soft-tissue sarcoma. Curr Oncol. 2020;27(suppl 1):17-23. doi:10.3747/co.27.5407 32174754 PMC7050043

[5] Ray-Coquard I, Cropet C, Van Glabbeke M, ; European Organization for Research and Treatment of Cancer Soft Tissue and Bone Sarcoma Group. Lymphopenia as a prognostic factor for overall survival in advanced carcinomas, sarcomas, and lymphomas. Cancer Res. 2009;69(13):5383-5391. doi:10.1158/0008-5472.CAN-08-3845 19549917 PMC2775079

[6] US Department of Health and Human Services. Common terminology criteria for adverse events (CTCAE) version 5.0. Published online November 27, 2017. Accessed January 27, 2021. https://ctep.cancer.gov/protocoldevelopment/electronic_applications/docs/ctcae_v5_quick_reference_5x7.pdf

[7] Teck Seo S, Singh VA, Yasin NF. Preoperative lymphocyte count in relation to sarcoma prognosis. J Orthop Surg (Hong Kong). 2019;27(2):2309499019854957. doi:10.1177/2309499019854957 31221016

[8] Sorbye SW, Kilvaer T, Valkov A, . Prognostic impact of lymphocytes in soft tissue sarcomas. PLoS One. 2011;6(1):e14611. doi:10.1371/journal.pone.0014611 21298041 PMC3029277

[9] Fujii H, Arakawa A, Utsumi D, . CD8^+^ tumor-infiltrating lymphocytes at primary sites as a possible prognostic factor of cutaneous angiosarcoma. Int J Cancer. 2014;134(10):2393-2402. doi:10.1002/ijc.28581 24243586

[10] Berghuis D, Santos SJ, Baelde HJ, . Pro-inflammatory chemokine-chemokine receptor interactions within the Ewing sarcoma microenvironment determine CD8^+^ T-lymphocyte infiltration and affect tumour progression. J Pathol. 2011;223(3):347-357. doi:10.1002/path.2819 21171080

[11] Zaorsky NG, Churilla TM, Egleston BL, . Causes of death among cancer patients. Ann Oncol. 2017;28(2):400-407. doi:10.1093/annonc/mdw604 27831506 PMC5834100

[12] Terrones C, Specht L, Maraldo MV, Lundgren J, Helleberg M. Lymphopenia after radiotherapy and risk of infection. Open Forum Infec Dis. 2017;4(suppl_1):S702. doi:10.1093/ofid/ofx163.1882

[13] Grossman SA, Ellsworth S, Campian J, . Survival in patients with severe lymphopenia following treatment with radiation and chemotherapy for newly diagnosed solid tumors. J Natl Compr Canc Netw. 2015;13(10):1225-1231. doi:10.6004/jnccn.2015.0151 26483062 PMC4778429

[14] Tolaney SM, Partridge AH, Sheib RG, Burstein HJ, Winer EP. *Pneumocystis carinii* pneumonia during dose-dense chemotherapy for breast cancer. J Clin Oncol. 2006;24(33):5330-5331. doi:10.1200/JCO.2006.08.1083 17114668

[15] Waks AG, Tolaney SM, Galar A, . *Pneumocystis jiroveci pneumonia* (PCP) in patients receiving neoadjuvant and adjuvant anthracycline-based chemotherapy for breast cancer: incidence and risk factors. Breast Cancer Res Treat. 2015;154(2):359-367. doi:10.1007/s10549-015-3573-2 26420402

[16] Tolaney SM, Najita J, Winer EP, Burstein HJ. Lymphopenia associated with adjuvant anthracycline/taxane regimens. Clin Breast Cancer. 2008;8(4):352-356. doi:10.3816/CBC.2008.n.041 18757263

