# Supplementary material for: Evaluation of Absolute Lymphocyte Count at Diagnosis and Mortality Among Patients With Localized Bone or Soft Tissue Sarcoma
Source: JAMA Netw Open. 2021 Mar 5;4(3):e210845. doi: 10.1001/jamanetworkopen.2021.0845 (PMC7936255; doi:10.1001/jamanetworkopen.2021.0845)
Supplement: Supplement. — eTable 1. Multivariate Cox Regression Analysis of the Association Between Minimum ALC and Overall Survival eTable 2. Multivariate Cox Regression Analysis of the Association Between Minimum ALC, Stratified by Lymphopenia Grade, and Overall Survival [file jamanetwopen-e210845-s001.pdf]

Supplementary Online Content

Brewster R, Purington N, Henry S, Wood D, Ganjoo K, Bui N. Evaluation of absolute lymphocyte count at diagnosis and mortality among patients with localized bone or soft tissue sarcoma. *JAMA Netw Open.* 2021;4(3):e210845. doi:10.1001/jamanetworkopen.2021.0845

eTable 1. Multivariate Cox Regression Analysis of the Association Between Minimum ALC and Overall Survival
eTable 2. Multivariate Cox Regression Analysis of the Association Between Minimum ALC, Stratified by Lymphopenia Grade, and Overall Survival

This supplementary material has been provided by the authors to give readers additional information about their work.

© 2021 Brewster R et al. *JAMA Network Open.*

eTable 1. Multivariate Cox Regression Analysis of the Association Between Minimum ALC and Overall Survival

**Lab Value**	**Patients (n)**	**Hazard Ratio (95% CI)**	***P* Value**
Minimum WBC			
Low	78	0.61 (0.33-1.12)	0.11
Normal (>4 K/uL, <11 K/uL)	527	1	
High	29	1.20 (0.65-2.21)	0.55
Minimum ANC			
Low	78	0.58 (0.20-1.68)	0.32
Normal (>1.7 K/uL, <6.7 K/uL)	527	1	
High	29	1.29 (0.90-1.86)	0.16
Minimum ALC			
Low	281	1.82 (1.39-2.40)	**<0.001**
Normal (>1 K/uL,<3 K/uL)	335	1	
High	18	1.19 (0.52-2.74)	0.16
Tumor Grade*			
1	88	1	
2	81	0.97 (0.47-1.98)	0.93
3	85	3.82 (2.13-6.85)	**<0.001**
4	163	3.38 (1.96-5.85)	**<0.001**
Age at Diagnosis		1.02 (1.01-1.02)	**<0.001**

© 2021 Brewster R et al. *JAMA Network Open.*

eTable 2. Multivariate Cox Regression Analysis of the Association Between Minimum ALC, Stratified by Lymphopenia Grade, and Overall Survival

Lab Value	Patients (n)	Hazard Ratio (95% CI)	*P* Value
Minimum WBC			
Low	78	0.56 (0.30-1.04)	0.11
Normal (>4 K/uL, <11 K/uL)	527	1	
High	29	1.20 (0.65-2.21)	0.55
Minimum ANC			
Low	78	0.56 (0.19-1.61)	0.32
Normal (>1 K/uL,<3 K/uL)	527	1	
High	29	1.30 (0.90-1.87)	0.16
Minimum ALC			
Low, Grade 1/2 (>0.5 K/uL, ≦1 K/uL)	181	1.60 (1.18-2.18)	**0.002**
Low, Grade 3/4 (≦0.5 K/uL)	100	2.44 (1.68-3.55)	**<0.001**
Normal (>1 K/uL,<3 K/uL)	335	1	
High	18	1.20 (0.52-2.75)	0.16
Tumor Grade*			
1	88	1	
2	81	1.02 (0.50-2.11)	0.93
3	85	3.99 (2.22-7.16)	**<0.001**
4	163	3.45 (2.00-5.96)	**<0.001**
Age at Diagnosis		1.02 (1.01-1.02)	**<0.001**

© 2021 Brewster R et al. *JAMA Network Open.*
